# Supplementary material for: MC1R loss-of-function is associated with accelerated Parkinson’s disease motor decline
Source: medRxiv. 2025 Dec 29:2025.12.26.25343003. Preprint. [Version 1] doi: 10.64898/2025.12.26.25343003 (PMC12772648; doi:10.64898/2025.12.26.25343003)
Supplement: Supplement 1 [file NIHPP2025.12.26.25343003v1-supplement-1.pdf]

	Sporadic PD	r Variant *MC1R* PD	R Variant *MC1R* PD
N	185	150	102
MC1R compound heterozygotes	0	22 (14.67)	42 (41.2)
MC1R homozygotes	0	13 (8.67)	4 (3.92)
Age at baseline (years)	65.2 (9.88)	61.3 (10.1)	61.2 (9.98)
Years since original diagnosis	0.76 (1.51)	0.63 (0.73)	0.62 (0.74)
Age at PD onset	64.4 (9.96)	60.7 (10.1)	60.6 (10.1)
Male	112 (60.5)	103 (68.7)	66 (64.7)
Race			
White	168 (90.8)	133 (88.7)	96 (94.1)
Asian	1 (0.54)	7 (4.67)	0
Black	4 (2.16)	2 (1.33)	1 (0.98)
Multiracial	7 (3.78)	4 (2.65)	2 (1.97)
Unknown	5 (2.70)	4 (2.65)	3 (2.95)
Hispanic or Latino ethnicity	19 (10.3)	4 (2.65)	3 (2.94)
Relatives with PD			
Parent	43 (23.2)	13 (8.67)	11 (10.8)
Other	46 (24.9)	24 (16.0)	21 (20.6)
α-Synuclein SAA status			
Positive	112	138	97
Negative	9	5	3
Unknown	64	7	2
α-Synuclein SAA positivity	92.6	96.5	95.1
History of melanoma	1 (0.54)	4 (2.65)	1 (0.98)
MDS-UPDRS I score at baseline	4.41 (3.18)	4.63 (3.45)	4.51 (3.34)
MDS-UPDRS III score at baseline	22.7 (9.6)	20.4 (8.99)	19.3 (8.76)
MoCA score at baseline	26.8 (2.35)	26.9 (2.75)	27.5 (2.30)
DAT-SPECT SBR at baseline			
Caudate	1.93 (1.63, 2.28)	1.99 (1.65, 2.32)	2.02 (1.68, 2.36)
Putamen	0.77 (0.63, 0.91)	0.84 (0.67, 0.99)	0.87 (0.65, 1.00)

Supplemental Table 1. Baseline demographic and clinical characteristics for sporadic and *MC1R* PD stratified by *MC1R* variant penetrance
Data is shown as n (%), %, mean (standard deviation), or median (interquartile range). Statistical analysis comparing group characteristics was not performed. A single participant may be included in both the r Variant and R Variant groups due to the presence of compound heterozygotes. Abbreviations: PD, Parkinson's disease; MC1R, melanocortin 1 receptor; SAA, seed amplification assay; MDS-UPDRS I and III, Movement Disorder Society Unified Parkinson's Disease Rating Scale Part I and III; MoCA, Montreal Cognitive Assessment; DAT-SPECT, dopamine transporter imaging with single-photon emission computed tomography; SBR, specific binding ratio.

	Sporadic PD	Single Heterozygote *MC1R* PD	Compound Heterozygote *MC1R* PD	Homozygote *MC1R* PD
N	185	139	64	17
r variant *MC1R* PD	0	83 (59.7)	22 (34.4)	13 (76.5)
R variant *MC1R* PD	0	56 (40.3)	42 (65.6)	4 (23.5)
Age at baseline (years)	65.2 (9.88)	61.2 (10.1)	61.3 (10.2)	64.2 (11.3)
Years since original diagnosis	0.76 (1.51)	0.66 (0.86)	0.60 (0.54)	0.39 (0.28)
Age at PD onset	64.4 (9.96)	60.5 (10.1)	60.7 (10.1)	63.8 (11.3)
Male	112 (60.5)	97 (69.8)	44 (68.8)	7 (41.2)
Race				
White	168 (90.8)	128 (92.1)	57 (89.1)	14 (82.4)
Asian	1 (0.54)	1 (0.72)	3 (4.70)	3 (17.6)
Black	4 (2.16)	3 (2.15)	0	0
Multiracial	7 (3.78)	4 (2.88)	2 (3.10)	0
Unknown	5 (2.70)	3 (2.15)	2 (3.10)	0
Hispanic or Latino ethnicity	19 (10.3)	1 (0.72)	3 (4.70)	0
Relatives with PD				
Parent	43 (23.2)	13 (9.35)	7(10.9)	1 (5.88)
Other	46 (24.9)	27 (19.4)	12 (18.8)	0
α-Synuclein SAA status				
Positive	112	126	60	17
Negative	9	7	1	0
Unknown	64	6	3	0
α-Synuclein SAA positivity	92.6	94.7	98.4	100
History of melanoma	1 (0.54)	4	1	0
MDS-UPDRS I score at baseline	4.41 (3.18)	4.70 (3.62)	4.48 (2.93)	4.00 (3.22)
MDS-UPDRS III score at baseline	22.7 (9.6)	20.2 (9.15)	19.1 (7.78)	20.7 (10.6)
MoCA score at baseline	26.8 (2.35)	27.1 (2.60)	27.4 (2.52)	27.2 (2.84)
DAT-SPECT SBR at baseline				
Caudate	1.93 (1.63, 2.28)	2.00 (1.67, 2.36)	2.01 (1.68, 2.27)	2.00 (1.44, 2.48)
Putamen	0.77 (0.63, 0.91)	0.85 (0.65, 1.00)	0.87 (0.65, 0.99)	0.84 (0.67, 0.98)

Supplemental Table 2. Baseline demographic and clinical characteristics for sporadic and *MC1R* PD stratified by *MC1R* genotype

Data is shown as n (%), %, mean (standard deviation), or median (interquartile range). Statistical analysis comparing group characteristics was not performed. Abbreviations: PD, Parkinson's disease; MC1R, melanocortin 1 receptor; SAA, seed amplification assay; MDS-UPDRS I and III, Movement Disorder Society Unified Parkinson's Disease Rating Scale Part I and III; MoCA, Montreal Cognitive Assessment; DAT-SPECT, dopamine transporter imaging with single-photon emission computed tomography; SBR, specific binding ratio.

	Sporadic PD	R163Q/P PD	V60L PD	V92M/L PD	R151S/G/C PD	R160W PD	D294N/H PD	D84E PD	R142H PD
N	185	41	77	54	50	43	11	4	3
MC1R compound heterozygotes	0	17 (41.5)	27 (35.1)	0	13 (26.0)	3 (6.98)	2 (18.2)	2 (50.0)	0
MC1R homozygotes	0	4 (9.76)	5 (6.49)	4 (7.41)	2 (4.00)	2 (4.65)	0	0	0
Age at baseline (years)	65.2 (9.88)	61.2 (10.1)	61.9 (11.0)	60.4 (9.85)	62.8 (10.9)	62.2 (9.12)	57.5 (7.67)	56.8 (7.50)	58.3 (9.29)
Years since original diagnosis	0.76 (1.51)	0.61 (0.56)	0.70 (0.86)	0.52 (0.53)	0.61 (0.87)	0.66 (0.61)	0.58 (0.52)	0.29 (0.14)	0.75 (0.08)
Age at PD onset	64.4 (9.96)	60.6 (9.99)	61.2 (10.9)	59.9 (9.84)	62.2 (11.1)	61.5 (9.19)	56.9 (7.77)	56.5 (7.62)	57.6 (9.21)
History of melanoma	1 (0.54)	0.00	2 (2.60)	3 (5.56)	0.00	1 (2.32)	0	0	0.00
Male	112 (60.5)	29 (70.7)	54 (70.1)	36 (66.67)	29 (58.0)	29 (67.4)	7 (63.6)	3 (75.0)	3 (100)
Race									
White	168 (90.8)	31 (75.6)	72 (93.5)	47 (87.0)	50 (100)	39 (90.7)	9 (81.8)	4 (100)	3 (100)
Asian	1 (0.54)	6 (14.6)	0	4 (7.40)	0	0	0	0	0
Black	4 (2.16)	0	0	2 (3.70)	0	1 (2.32)	0	0	0
Multiracial	7 (3.78)	2 (4.90)	3 (3.90)	1 (1.90)	0	1 (2.32)	1 (9.10)	0	0
Unknown	5 (2.70)	2 (4.90)	2 (2.60)	0	0	2 (4.65)	1 (9.10)	0	0
Hispanic or Latino ethnicity	19 (10.3)	3 (7.32)	0	1	0	1 (2.32)	1 (9.10)	1 (25.0)	0
Relatives with PD									
Parent	43 (23.2)	6 (14.6)	6 (7.80)	3 (5.56)	8 (16.0)	2 (4.65)	1 (9.10)	1 (25.0)	0
Other	46 (24.9)	2 (4.90)	13 (16.90)	12 (22.2)	14 (28.0)	7 (16.3)	2 (18.2)	1 (25.0)	0
α-Synuclein SAA status									
Positive	112	37	70	50	47	41	10	4	3
Negative	9	2	3	1	2	1	0	0	0
Unknown	64	2	4	3	1	1	1	0	0
α-Synuclein SAA positivity	92.6	94.9	95.9	98	95.9	97.6	100	100	100
History of melanoma	1 (0.54)	0.00	2 (2.60)	3 (5.56)	0.00	1 (2.32)	0	0	0.00
MDS-UPDRS I score at baseline	4.41 (3.18)	5.80 (3.51)	4.21 (3.04)	4.26 (3.99)	4.48 (3.59)	4.49 (3.15)	4.56 (2.13)	7.00 (5.66)	1.33 (1.53)
MDS-UPDRS III score at baseline	22.7 (9.6)	20.9 (9.07)	20.6 (8.81)	18.8 (9.10)	19.8 (9.49)	18.1 (7.54)	20.7 (9.02)	34.5 (6.36)	15.7 (7.09)
MoCA score at baseline	26.8 (2.35)	26.4 (2.39)	27.1 (2.84)	27.6 (2.79)	27.5 (2.38)	27.3 (2.58)	27.6 (2.40)	29.0 (1.41)	28.0 (2.00)
DAT-SPECT SBR at baseline									
Caudate	1.93 (1.63, 2.28)	2.04 (1.72, 2.43)	1.96 (1.53, 2.24)	2.00 (1.74, 2.20)	2.05 (1.75, 2.39)	2.00 (1.67, 2.33)	2.06 (1.49, 2.43)	1.76 (1.52, 1.99)	1.85 (1.60, 2.10)
Putamen	0.77 (0.63, 0.91)	0.93 (0.70, 1.06)	0.80 (0.60, 0.97)	0.82 (0.69, 0.99)	0.92 (0.66, 1.05)	0.82 (0.65, 0.92)	0.83 (0.62, 0.92)	0.78 (0.67, 0.89)	0.80 (0.69, 0.99)

Supplemental Table 3. Baseline demographic and clinical characteristics for sporadic and *MC1R* PD stratified by *MC1R* variant

Data is shown as n (%), %, mean (standard deviation), or median (interquartile range). Statistical analysis comparing group characteristics was not performed. A single participant may be included in several variant groups due to the presence of compound heterozygotes. Abbreviations: PD, Parkinson's disease; MC1R, melanocortin 1 receptor; SAA, seed amplification assay; MDS-UPDRS I and III, Movement Disorder Society Unified Parkinson's Disease Rating Scale Part I and III; MoCA, Montreal Cognitive Assessment; DAT-SPECT, dopamine transporter imaging with single-photon emission computed tomography; SBR, specific binding ratio.

		MDS-UPDRS I			MoCA			Caudate SBR			Putamen SBR		
Group	N	Slope, β (95% CI)	% diff	p-value	Slope, β (95% CI)	% diff	p-value	Slope, β (95% CI)	% diff	p-value	Slope, β (95% CI)	% diff	p-value
Sporadic PD	185	0.42 (0.34, 0.50)			-0.23 (-0.32, -0.13)			-0.13 (-0.15, -0.11)			-0.06 (-0.07, -0.06)		
MC1R PD vs. Sporadic PD	220 | 185	0.52, 0.10 (-0.01, 0.20)	24%	0.070	-0.21, 0.02 (-0.09, 0.14)	-10%	0.690	-0.13, 0.00 (-0.02, 0.03)	0%	0.908	-0.06, 0.00 (-0.01, 0.01)	0%	0.827
r variant *MC1R* PD	150 | 185	0.49, 0.07 (-0.05, 0.19)	17%	0.225	-0.30, -0.07 (-0.22, 0.07)	30%	0.324	-0.13, 0.00 (-0.03, 0.02)	0%	0.868	-0.06, 0.00 (-0.02, 0.01)	0%	0.682
R variant *MC1R* PD	102 | 185	0.55, 0.13 (0.00, 0.16)	31%	0.055	-0.13, 0.10 (-0.03, 0.23)	-30%	0.143	-0.12, 0.01 (-0.02, 0.04)	-8%	0.707	-0.06 0.00 (-0.02, 0.02)	0%	0.915
Single Heterozygote *MC1R* PD	139 | 185	0.49, 0.07 (-0.04, 0.19)	17%	0.202	-0.19, 0.04 (-0.09, 0.16)	-17%	0.575	-0.13, 0.00 (-0.03, 0.03)	0%	0.925	-0.06, 0.00 (-0.01, 0.02)	0%	0.780
Compound Heterozygote *MC1R* PD	64 | 185	0.50, 0.08 (-0.08, 0.23)	19%	0.352	-0.18, 0.05 (-0.13, 0.23)	-22%	0.596	-0.12, 0.01 (-0.02, 0.04)	-8%	0.620	-0.07, -0.01 (-0.03, 0.01)	17%	0.315
Homozygote *MC1R* PD	17 | 185	0.79, 0.37 (0.12, 0.62)	88%	**0.003**	-0.40, -0.17 (-0.46, 0.12)	74%	0.251	-0.13, 0.00 (-0.06, 0.05)	0%	0.942	-0.05, 0.01 (-0.02, 0.03)	-17%	0.668
R163Q/P PD	41 | 185	0.52, 0.10 (-0.08, 0.28)	24%	0.273	-0.08, 0.15 (-0.05, 0.35)	-65%	0.131	-0.13, 0.00 (-0.03, 0.04)	0%	0.821	-0.06, 0.00 (-0.02, 0.02)	0%	0.665
V60L PD	77 | 185	0.47, 0.05 (-0.09, 0.19)	12%	0.487	-0.13, 0.10 (-0.06, 0.26)	-43%	0.224	-0.12, 0.01 (-0.03, 0.04)	-8%	0.745	-0.06, 0.00 (-0.02, 0.02)	0%	0.972
V92M/L PD	54 | 185	0.47, 0.05 (-0.15, 0.24)	12%	0.650	-0.19, 0.04 (-0.18, 0.26)	-17%	0.741	-0.16, -0.03 (-0.08, 0.01)	23%	0.183	-0.06, 0.00 (-0.03, 0.02)	0%	0.771
R151S/G/C PD	50 | 185	0.53, 0.11 (-0.04, 0.26)	26%	0.157	-0.36, -0.13 (-0.30, 0.04)	-57%	0.140	-0.12, 0.01 (-0.02, 0.05)	-8%	0.386	-0.07, -0.01 (-0.03, 0.02)	17%	0.627
R160W PD	43 | 185	0.45, 0.03 (-0.14, 0.20)	7%	0.726	-0.25, -0.02 (-0.20, 0.17)	9%	0.852	-0.12, 0.01 (-0.03, 0.06)	-8%	0.566	-0.05, 0.01 (-0.01, 0.03)	-17%	0.498
D294N/H PD	11 | 185	0.57, 0.15 (-0.22, 0.52)	36%	0.431	-0.13, 0.10 (-0.32, 0.53)	-43%	0.627	-0.15, -0.02 (-0.10, 0.06)	15%	0.681	-0.06, 0.00 (-0.05, 0.04)	0%	0.915
D84E PD	4 | 185	0.59, 0.17 (-0.63, 0.96)	40%	0.678	0.15, 0.38 (-0.46, 1.22)	-165%	0.374	-0.07, 0.06 (-0.04, 0.16)	-46%	0.241	-0.07, -0.01 (-0.09, 0.08)	17%	0.878
R142H PD	3 | 185	1.02, 0.60 (0.03, 1.17)	143%	**0.039**	-0.20, 0.03 (-0.57, 0.62)	-13%	0.932	-0.14, -0.01 (-0.12, 0.10)	8%	0.830	-0.02, 0.04 (-0.04, 0.11)	-67%	0.359
LRRK2 PD vs. Sporadic PD	84 | 185	0.26, -0.16 (-0.30, -0.01)	-38%	**0.040**	-0.04, 0.19 (0.02, 0.36)	-83%	**0.027**	-0.12, 0.01 (-0.03, 0.05)	-8%	0.647	-0.06, 0.00 (-0.02, 0.02)	0%	0.812
GBA PD vs. Sporadic PD	43 | 185	0.51, 0.09 (-0.13, 0.31)	21%	0.413	-0.20, 0.03 (-0.20, 0.26)	-13%	0.803	-0.15, -0.02 (-0.08, 0.04)	15%	0.509	-0.07, -0.01 (-0.04, 0.01)	14%	0.319
MC1R + *LRRK2* PD vs. Sporadic PD	187 | 185	0.32, -0.10 (-0.23, 0.03)	-24%	0.138	-0.13, 0.10 (-0.04, 0.24)	-43%	0.160	-0.11, 0.02 (-0.01, 0.05)	-15%	0.124	-0.05, 0.01 (-0.01, 0.03)	-14%	0.183
MC1R + *GBA* PD vs. Sporadic PD	83 | 185	0.43, 0.01 (-0.15, 0.17)	2%	0.894	-0.26, -0.03 (-0.21, 0.15)	13%	0.725	-0.13, 0.00 (-0.04, 0.04)	0%	0.882	-0.05, 0.01 (-0.02, 0.03)	-14%	0.577
MC1R + *LRRK2* PD vs. *LRRK2* PD	187 | 84	0.32, 0.06 (-0.09, 0.21)	23%	0.429	-0.13, -0.09 (-0.26, 0.08)	225%	0.312	-0.11, 0.01 (-0.03, 0.06)	-8%	0.538	-0.05, 0.01 (-0.02, 0.03)	-14%	0.552
MC1R + *GBA* PD vs. *GBA* PD	83 | 43	0.43, -0.08 (-0.33, 0.16)	-16%	0.515	-0.26, -0.06 (-0.32, 0.19)	30%	0.615	-0.13, 0.02 (-0.05, 0.09)	-13%	0.531	-0.05, 0.02 (-0.02, 0.05)	-28%	0.308

Supplemental Table 4. Comparison of the rate of change in MDS-UPDRS I, MoCA, and DAT-SPECT SBR between participants with sporadic, *MC1R*, *LRRK2*, *GBA*, *MC1R* + *LRRK2*, and *MC1R* + *GBA* PD

Slopes (unit per year) were estimated from linear mixed models. Fixed effects included time * genetic group, baseline MDS-UPDRS III, baseline age, years since original diagnosis to baseline, sex, race, ethnicity, and LEDD. Participant-level random intercepts and slopes with unstructured covariance, heteroscedastic by genetic group. % difference is relative to the direction of change of each assessment. MC1R PD, LRRK2 PD, and GBA PD were compared to sporadic PD in a single model while MC1R+LRRK2 PD and MC1R+GBA PD were compared to LRRK2 PD and GBA PD in separate models. Stratification by penetrance, genotype, and variant was performed in separate models using sporadic PD as the reference group. A single participant may be included in multiple groups due to the presence of compound heterozygotes. N refers to the comparison group | reference group. Abbreviations: PD, Parkinson's disease; MC1R, melanocortin 1 receptor; MDS-UPDRS I and III, Movement Disorder Society Unified Parkinson's Disease Rating Scale Part I and III; MoCA, Montreal Cognitive Assessment; DAT-SPECT, dopamine transporter imaging with single-photon emission computed tomography; SBR, specific binding ratio.

	Sporadic Prodromal PD	*MC1R* Prodromal PD
N	16	29
R163Q/P carriers	----	6 (20.7)
V60L carriers	----	7 (24.1)
V92M/L carriers	----	6 (20.7)
R151S/G/C carriers	----	6 (20.7)
R160W carriers	----	7 (24.1)
D294N/H carriers	----	0
D84E carriers	----	0
R142H carriers	----	3 (10.3)
MC1R compound heterozygotes	----	6 (20.7)
MC1R homozygotes	----	2 (6.89)
Phenoconverters	4 (25.0)	18 (62.1)
Age at baseline (years)	68.8 (6.20)	69.6 (5.82)
Average follow-up (years)	6.14 (3.16)	8.31 (2.72)
Male	16 (100)	22 (75.9)
Race		
White	14 (87.5)	27 (93.1)
Black	1 (6.25)	1 (3.45)
Unknown	1 (6.25)	1 (3.45)
Hispanic or Latino ethnicity	16 (100)	22 (75.9)
Relatives with PD		
Parent	2 (12.5)	1 (3.45)
Other	0	2 (6.90)
History of melanoma	0	0

Supplemental Table 5. Demographic characteristics for PPMI participants with sporadic and *MC1R* prodromal PD

Data is shown as n (%) or mean (standard deviation). Statistical analysis comparing group characteristics was not performed. A single participant may be included in several carrier groups due to the presence of compound heterozygotes. Abbreviations: PD, Parkinson's disease; MC1R, melanocortin 1 receptor
